# Prenatal ultrasound in fetuses with polycystic kidney appearance — expanding the diagnostic algorithm

**DOI:** 10.1007/s00404-022-06814-8

**Published:** 2022-10-31

**Authors:** Corinna Simonini, Eva-Maria Fröschen, Jennifer Nadal, Brigitte Strizek, Christoph Berg, Annegret Geipel, Ulrich Gembruch

**Affiliations:** 1 Department of Obstetrics and Prenatal Medicine, University Hospital Bonn, Venusberg-Campus 1, 53127 Bonn, Germany; 2 Department of Medical Biometry, Informatics, and Epidemiology (IMBIE), University Hospital Bonn, Venusberg-Campus 1, 53127 Bonn, Germany; 3 Division of Prenatal Medicine, Department of Obstetrics and Gynecology, University of Cologne, Cologne, Germany

**Keywords:** ARPKD, ADPKD, Hyperechogenic kidneys, Ciliopathies, Corticomedullary differentiation

## Abstract

**Purpose:**

Report on the diagnosis of prenatally detected fetal kidneys with bilateral polycystic appearance in a single center between 1999 and 2020 with special focus on renal morphology and biometry, amniotic fluid and extrarenal findings and proposal for an diagnostic algorithm.

**Methods:**

Retrospective observational study including pregnancies with prenatally detected kidneys with bilateral polycystic appearance (*n* = 98). Cases and outcomes were compared according to prenatal findings with special focus on renal morphology, amount of amniotic fluid, and presence of extrarenal abnormalities.

**Results:**

Most frequent diagnoses were autosomal recessive polycystic kidney disease (ARPKD, 53.1%), Meckel–Gruber syndrome (MKS, 17.3%) and autosomal dominant polycystic kidney disease (ADPKD, 8.2%). Other diagnoses included: Joubert-, Jeune-, McKusick–Kaufman- and Bardet–Biedl syndrome, overgrowth syndromes, Mainzer–Saldino syndrome and renal tubular dysgenesis.

Renal abnormalities most frequently observed were hyperechogenic parenchyma, kidney enlargement, changes of corticomedullary differentiation and cystic changes of various degree. Oligo- and anhydramnios were mainly seen in ARPKD, RTD and second-trimester MKS. Extrarenal findings included skeletal (35.7%) and cardiac (34.7%) abnormalities as well as abnormalities of the central nervous system (27.6%).

**Conclusion:**

Gestational age at manifestation, kidney size, visibility of cysts, echogenicity, amniotic fluid volume, and the presence of associated extrarenal malformations allow to differentiate between the most frequent underlying diseases presenting with bilateral polycystic kidneys on prenatal ultrasound by following a diagnostic algorithm.

## What does this study add to the clinical work?

**Table:** 

In this study we present one of the largest series of prenatally diagnosed fetal kidneys with polycystic appearance. It adds valuable information on kidney morphology as well as extrarenal findings of different diseases presenting with fetal kidneys with polycystic appearance and hereby expands previously described diagnostic algorithms.	

## Introduction

Differential diagnosis of fetal cystic kidney disease is often challenging and there is continuing need for studies on genotype–phenotype correlations in different underlying diseases. Whereas some of those present with very distinct and specific additional abnormalities affecting multiple organ systems, others present with only subtle ultrasound features, a heterogeneous phenotypic expression or no extrarenal abnormalities at all. The amount of amniotic fluid, which is one of the crucial prognostic factors, can be helpful in making a differential diagnosis, but is not always specific [1, 2].

To a great extent, cystic kidneys are part of the broad spectrum of ciliopathies that may manifest either predominantly hepatorenally, or affect multiple organ systems of the human body due to the ubiquitous presence of primary cilia in various organ tissues [2, 3]. Whereas autosomal recessive polycystic kidney disease (ARPKD) and autosomal dominant polycystic kidney disease (ADPKD) are representatives of ciliopathies with mainly hepatorenal predilection, multisystem ciliopathies such as Jeune syndrome, Joubert syndrome (JS) or Meckel–Gruber syndrome (or Meckel syndrome, MKS) are on the other end of the spectrum and may also affect the skeletal, central nervous or other organ systems [2]. To a smaller extent, polycystic and hyperechogenic kidneys may be found in overgrowth syndromes such as Beckwith–Wiedemann (BWS) or Simpson–Golabi–Behmel syndrome (SGBS) [4] or in rare genetic diseases such as renal tubular dysgenesis (RTD) [5].

When confronted with polycystic kidneys on prenatal ultrasound, the varying spectrum of severity of the different underlying diseases, which ranges from favorable (such as in ADPKD, McKusick–Kaufman syndrome or Bardet–Biedl syndrome [MKKS/BBS]) to severe (such as in ARPKD) and to even fatal (such as in MKS) has to be considered [2].

Hence, correct interpretation of fetal cystic kidney changes is crucial. The aim of this study was to retrospectively analyze our cases of bilateral polycystic kidneys with special focus on renal morphology and biometry together with clinical and genetic findings, to suggest additional sonographic criteria to help differentiate between different diseases by prenatal ultrasound.

## Methods

This was a retrospective observational study including the data of pregnancies booked at two tertiary level referral centers of Obstetrics and Prenatal Medicine (Bonn and Cologne, Germany) in the period between 1999 and 2020. Referrals to our centers represent a mixed low- and high-risk population and are sent for targeted ultrasound examination or evaluation of suspected fetal anomalies. Most cases included in this study were sent because of oligo- or anhydramnios and/or hyperechogenic and cystic kidneys. All women received a detailed fetal anomaly scan including fetal echocardiography using high-resolution ultrasound equipment, followed by extensive multidisciplinary counseling.

We reviewed our data on the specific sonographic findings with special focus on fetal kidney morphology and biometry in different diseases, amount of amniotic fluid (AF), presence of extrarenal findings, genetic results as well as pregnancy outcome and long-term outcome of affected children, if available. Outcomes were obtained from our perinatal database, neonatal records or autopsy findings.

Regarding ultrasound evaluation of the fetal kidneys, we aimed to assess kidney size, echogenicity as well as corticomedullary differentiation (CMD), size and localization of cysts and changes of the renal pelvicalyceal system. To define hyperechogenicity of the renal parenchyma, liver parenchyma was used for comparison [6]. Longitudinal kidney diameters were used to assess the degree of enlargement of the kidneys [7]. Cases with only unilateral kidney changes, urinary tract obstruction as well as drug-induced nephropathies were excluded from this study. Some of the cases included have been reported previously [1, 8]. Statistical analysis was performed using SAS 9.4 (2002–2012 by SAS Institute Inc., Cary, NC, USA). Outcomes were quantified as means with standard deviation ( ± SD) and the range. All patients had given written informed consent for data collection, analysis and their use for research. The Ethics Committees of the Universities of Bonn and Cologne do not request formal approval for anonymized retrospective analysis of clinical data.

## Results

Between January of 1999 and December of 2020, 98 fetuses were diagnosed with bilaterally polycystic kidneys. Gestational age at initial presentation (mean: 24 + 2 weeks’; range: 11 + 1–38 + 3 weeks’) is shown in Table 1. Initial diagnosis before 22 + 0 weeks’ gestation was made in 43 cases (43.9%). 17 suffered from ARPKD, 14 from MKS, 5 from JS, 2 from ADPKD, 2 from SGBS; in two cases final diagnosis was unclear and there was one case of BWS.

**Table 1:** Characteristics of fetuses with prenatally diagnosed polycystic kidney appearance (*n* = 98)

Diagnosis	Mean GA at diagnosis (range)	Amniotic fluid at initial presentation	Sex	Outcome	Kidney morphology			
					Hyperechogenic parenchyma	CMD	Cysts	Hydronephrosis
All (*n* = 98)	24 + 2 (11 + 1–38 + 3)	Anhydramnios (*n* = 22) Oligohydramnios (*n* = 36) Normal (*n* = 36) Polyhydramnios (*n* = 4)	Male (*n* = 53) Female (*n* = 41) Unknown (*n* = 4)	Live-born (*n* = 33) Perinatal death (*n* = 3) TOP (*n* = 57) IUD (*n* = 4) Lost to follow-up (*n* = 1)				
ARPKD (*n* = 52)	25 + 2 (16 + 3–38 + 3)	Anhydramnios (*n = *16) Oligohydramnios (*n* = 28) Normal (*n* = 8)	Male (*n* = 27) Female (*n* = 25)	TOP (n = 25) Perinatal death (*n* = 2) IUD (*n* = 2) Live-born (*n* = 23) Lost to follow-up (*n* = 1)	Yes (*n* = 36) No (*n* = 16)	Reduced/absent (*n* = 48) Reverse (*n* = 4) [calcifications (*n* = 1)]	Yes (*n* = 31) No (*n* = 21)	No (*n* = 52)
ADPKD (*n* = 8)	26 + 3 (20 + 5–32 + 6)	Anhydramnios (*n* = 1) normal (*n* = 7)	Male (*n* = 3) Female (*n* = 5)	TOP (*n* = 2) Live-born (*n* = 6)	Yes (*n* = 8)	Reduced/absent (*n* = 2) Normal (*n* = 2) Enhanced (*n* = 4)	Yes (*n* = 3) No (*n* = 5)	No (*n* = 8)
MKS (*n* = 17)	19 + 3 (11 + 1–35 + 3)	Anhydramnios (*n* = 3)^c^ Oligohydramnios (*n* = 5)^c^ Normal (*n* = 9)^c^	Male (*n* = 9) Female (*n* = 5) Unknown (*n* = 3)	TOP (*n* = 15) Perinatal death (*n* = 1) IUD (*n* = 1)	Yes (*n* = 14) No (*n* = 3)	Absent (*n* = 14)^a^ Normal (*n* = 3)	Yes (*n* = 17)	No (*n* = 17)
Joubert^b^ (*n* = 5)	20 + 0 (16 + 3–21 + 0)	Normal (*n* = 5)	Male (*n* = 5)	TOP (*n* = 5)	Yes (*n* = 1) No (*n* = 3)	Normal (*n* = 2) Absent (*n* = 2)	Yes (*n* = 2) N o (*n* = 2)	No (n = 4)
MKKS/BBS (*n* = 4)	27 + 6 (22 + 4–37 + 2)	Normal (*n* = 4)	Male (*n* = 1) Female (*n* = 3)	TOP (*n* = 2) Live-born (*n* = 2)	Yes (*n* = 4)	Absent (*n* = 4)	Yes (*n* = 4)	Yes (*n* = 2) No (*n* = 2)
SGBS (*n* = 3)	20 + 4 (17 + 4–22 + 6)	Normal (*n* = 1) Polyhydramnios (*n* = 2)	Male (*n* = 3)	TOP (*n* = 2) Live-born (*n* = 1)	Yes (*n* = 2) No (*n* = 1)	Absent (*n* = 3)	Yes (*n* = 3)	Yes (*n* = 1) No (*n* = 2)
Jeune (*n* = 2)	24 + 5 (23 + 2–26 + 2)	Normal (*n* = 2)	Male (*n* = 1) Female (*n* = 1)	TOP (*n* = 2)	No (*n* = 2)	Absent (*n* = 2)	Yes (*n* = 2)	No (*n* = 2)
Mainzer–Saldino (*n* = 1)	30 + 3	Normal (*n* = 1)	Male (*n* = 1)	Live-born (*n* = 1)	Yes (*n* = 1)	Absent (*n* = 1)	Yes (*n* = 1)	No (*n* = 1)
BWS (*n* = 2)	24 + 6 (21 + 0–28 + 6)	Polyhydramnios (*n* = 2)	Male (*n* = 1) Female (*n* = 1)	TOP (*n* = 1) Live-born (*n* = 1)	Yes (*n* = 1) No (*n* = 1)	Absent (*n* = 2)	Yes (*n* = 2)	No (*n* = 2)
RTD (*n* = 1)	31 + 4	Anhydramnios (*n* = 1)	Female (*n* = 1)	TOP (*n* = 1)	Yes (*n* = 1)	Reduced/absent (*n* = 1)	No (*n* = 1)	No (*n* = 1)
Unknown (*n *= 3)	22 + 2 (20 + 2–26 + 0)	Anhydramnios (*n* = 1) Oligohydramnios (*n* = 2)	Male (*n* = 2) Unknown (*n* = 1)	TOP (*n* = 2) IUD (*n* = 1)	Yes (*n* = 2) No (*n* = 1)	Absent (*n* = 3)	Yes (*n* = 2) No (*n* = 1)	No (*n* = 3)

*ADPKD* autosomal dominant polycystic kidney disease, *ARPKD* autosomal recessive polycystic kidney disease, *BBS* Bardet-Biedl syndrome, *BWS* Beckwith-Wiedemann syndrome, *CMD* corticomedullary differentiation, *GA* gestational age, *IUD* intrauterine death, *MKKS* McKusick-Kaufman syndrome, *MKS* Meckel-Gruber syndrome (Meckel syndrome), *RTD* renal tubular dysgenesis, *SGBS* Simpson-Golabi-Behmel syndrome, *TOP* termination of pregnancy;

^a^Absent CMD from advanced 2nd trimester onwards

^b^Lack of image material in one case

^c^Fetuses initially presenting with oligo- or anhydramnios: mean GA at initial presentation 24 + 4 weeks’, all nine cases with normal AF at initial presentation presented before 20 weeks’ gestation

Six families had more than one pregnancy affected, which leaves 89 different families in total and parental consanguinity was observed in 12 families (13.5%).

ARPKD was diagnosed in 52 fetuses (53.1%), ADPKD in 8 fetuses (8.2%), MKS in 17 fetuses (17.3%), JS in 5 fetuses (5.1%), MKKS/BBS in 4 fetuses (4.1%), SGBS in 3 fetuses (3.1%), Jeune syndrome and BWS in 2 fetuses (2.0%), each, and there was one case (1%) of Mainzer–Saldino syndrome and renal tubular dysgenesis (RTD), each. In three fetuses (3.1%), the final diagnosis remained unclear. Karyotyping (either pre- or postnatally) was performed in 56 cases (57.1%) and abnormal genetic findings (found in 35.7%) are presented as part of the supplementary material. Of all eight cases of ADPKD, seven mothers (87.5%) also showed polycystic kidneys. In one of these cases, initial diagnosis in the mother was only made because of the sonographic findings in the fetus. One case (12.5%) showed de-novo mutation of PKD1.

### Ultrasound findings

#### Renal morphology and biometry

**Fig. 1:**
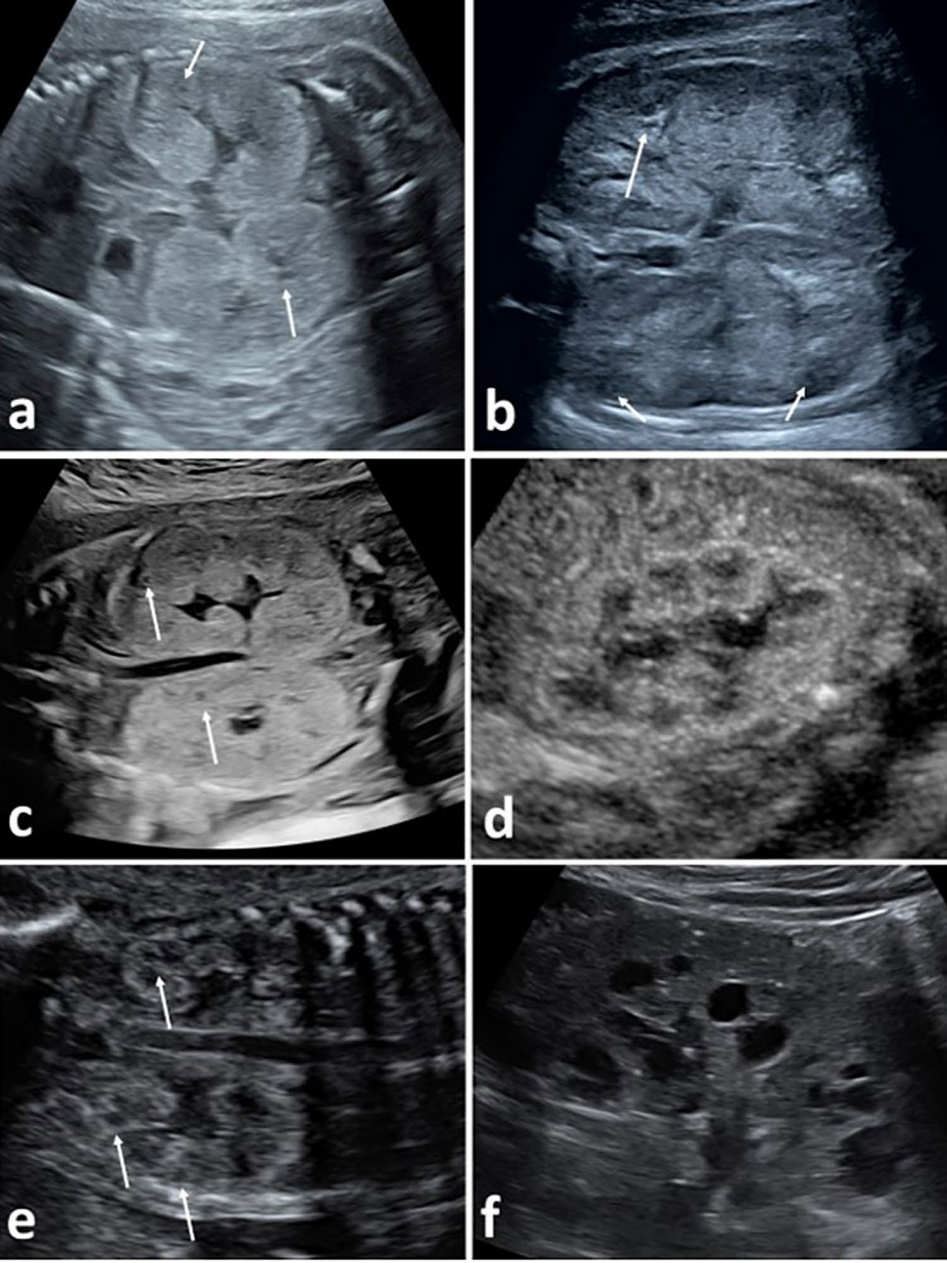
Prenatal ultrasound in autosomal recessive polycystic kidney disease (*ARPKD ***a**, **b**) and autosomal dominant polycystic kidney disease (*ADPKD*, **c**–**f**). **a** shows fetal kidneys in *ARPKD* at 23+3 weeks’: kidneys present enlarged, hyperechogenic and with absent corticomedullary differentiation (CMD); small cysts can be seen in the medulla (white arrow); cysts show a variable size from 1 to 9 mm and a relative increase in size with advancing gestational age; **b** shows the kidneys of another fetus with *ARPKD* at 30+5 weeks’: kidneys are enlarged with reverse *CMD* (hyperechogenic medulla and hypoechogenic cortex). In this case, cysts (white arrow) are predominantly located in the cortex, however in most cases of *ARPKD* they can be seen in both cortex and medulla; **c** shows kidneys of a fetus with *ADPKD* at 24+0 weeks’: in this case, kidneys are enlarged, hyperechogenic with absent corticomedullary differentiation (*CMD*) and small cysts (white arrows); **d** kidney of another fetus with *ADPKD* at 28+0 weeks’, showing enhanced CMD; **e** shows the kidneys of another fetus with *ADPKD* at 24+1 weeks’: *CMD* is preserved with visible cysts (white arrows) predominantly located in the cortex; **f** shows the kidney of the mother of the fetus in **e** with multiple cysts in both cortex and medulla.

**Fig. 2:**
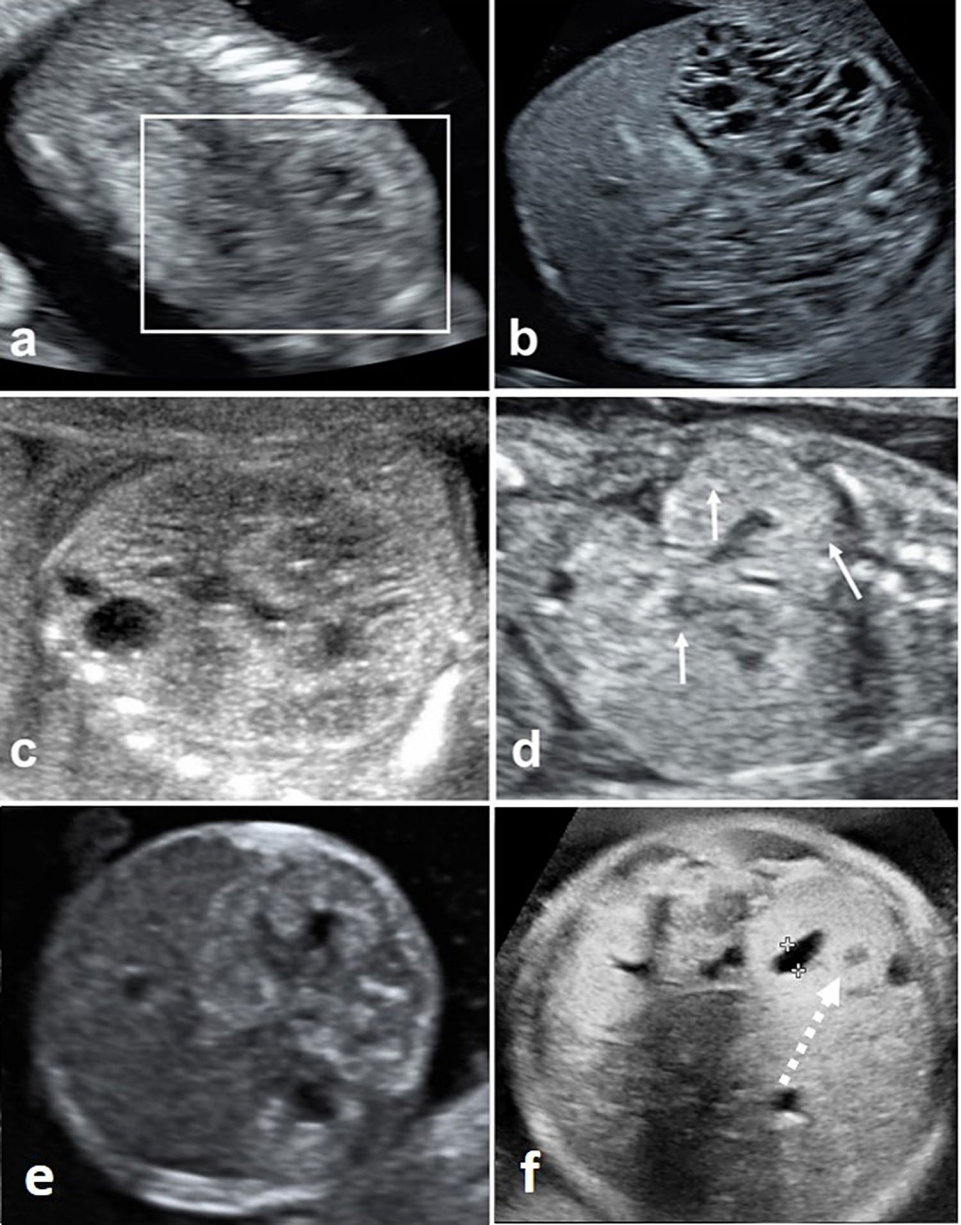
Prenatal ultrasound in fetuses with Meckel-Gruber syndrome (*MKS*, **a**–**c**) and Simpson–Golabi–Behmel syndrome (*SGBS*, **d**-**f**).** a** Shows a fetus with *MKS* at 12+0 weeks’: corticomedullary differentiation (*CMD*) appears premature or “too advanced” for the gestational age (hyperechogenic cortex, hypoechogenic medulla; white box); **b **shows the same fetus at 14+5 weeks’: both kidneys (axial view) present with multiple cysts of different size both in cortex and medulla (with accentuated occurrence in medulla, causing for the “mottled appearance”); **c** shows a different fetus at 19+4 weeks’ with enlarged kidneys presenting with absent *CMD* and cysts of various size interspersing the entire parenchyma; **d**–**e** show a fetus with *SGBS* in 17+4 weeks’, presenting with mildly enlarged, hyperechogenic kidneys showing absent corticomedullary differentiation (*CMD*, coronary and axial view) as well as small cysts (white arrows); **e** shows another fetus with SGBS at 31+3 weeks’, presenting with enlargend and hyperehogenic kidneys with absent *CMD* and small cysts (white arrow) as well as mild hydronephrosis (axial view, dotted white arrow)

**Fig. 3:**
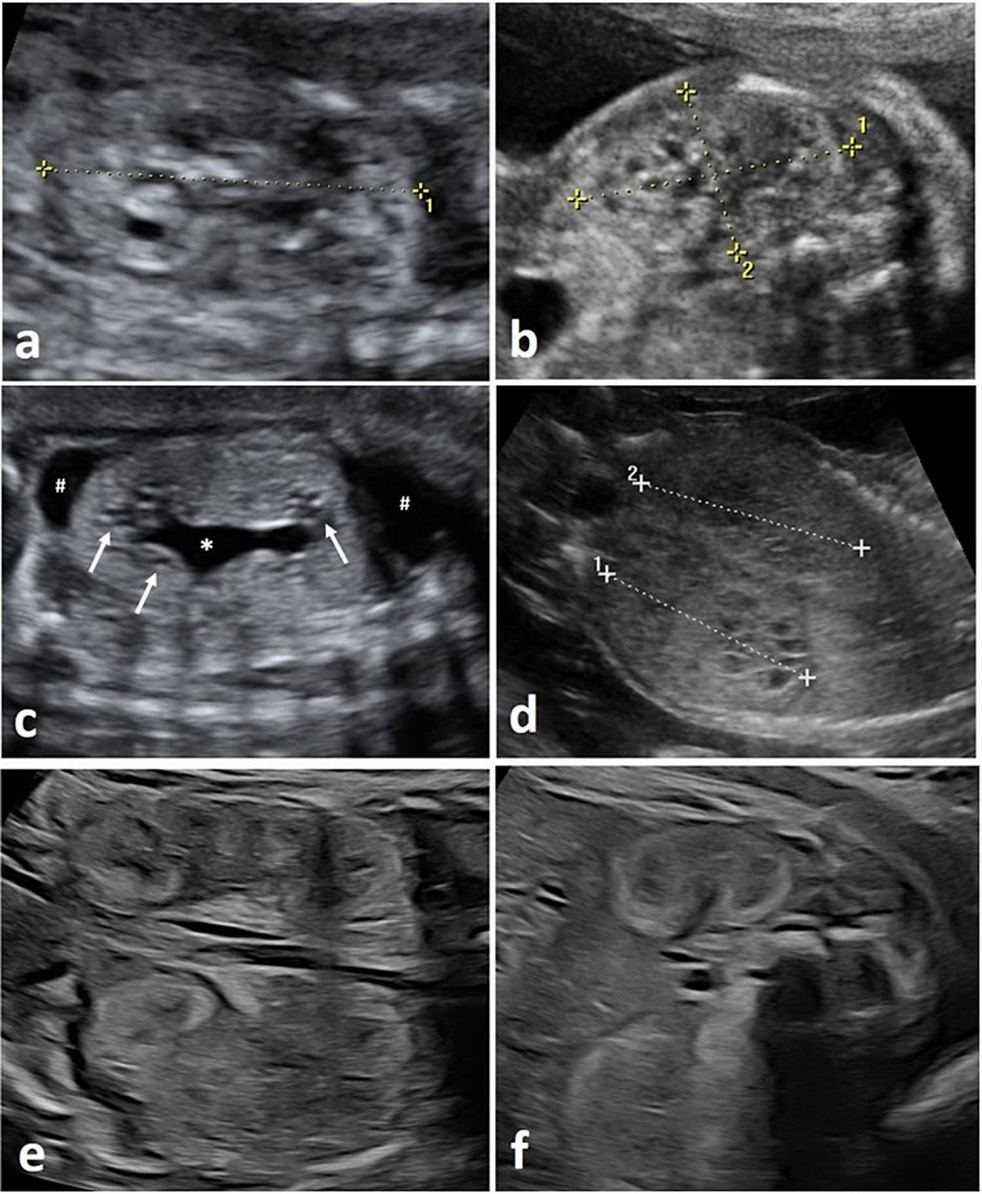
Prenatal ultrasound in fetuses with Joubert syndrome (J*S*), McKusick–Kaufman syndrome/Bardet–Biedl syndrome (*MKKS/BBS*), Jeune syndrome and renal tubular dysgenesis (*RTD*).** a**+**b** Show a fetus with JS in 20+6 weeks’: both kidneys present small cysts in both cortex and medulla; **c** shows a fetus with *MKKS/BBS* at 26+0 weeks’: kidneys are enlarged and hyperechogenic with absent *CMD*, small cysts (white arrows) as well as mild hydronephrosis (asterisk); additionally, this fetus showed mild ascites (number sign); **d** shows a fetus with Jeune syndrome in 23+2 weeks‘: kidneys present enlarged, isoechogenic with absent *CMD* and small cysts in both cortex and medulla; **e**+**f **show fetal kidneys in RTD at 32+1 weeks’: kidneys present enlarged, hyperechogenic and with reduced *CMD*

**Fig. 4:**
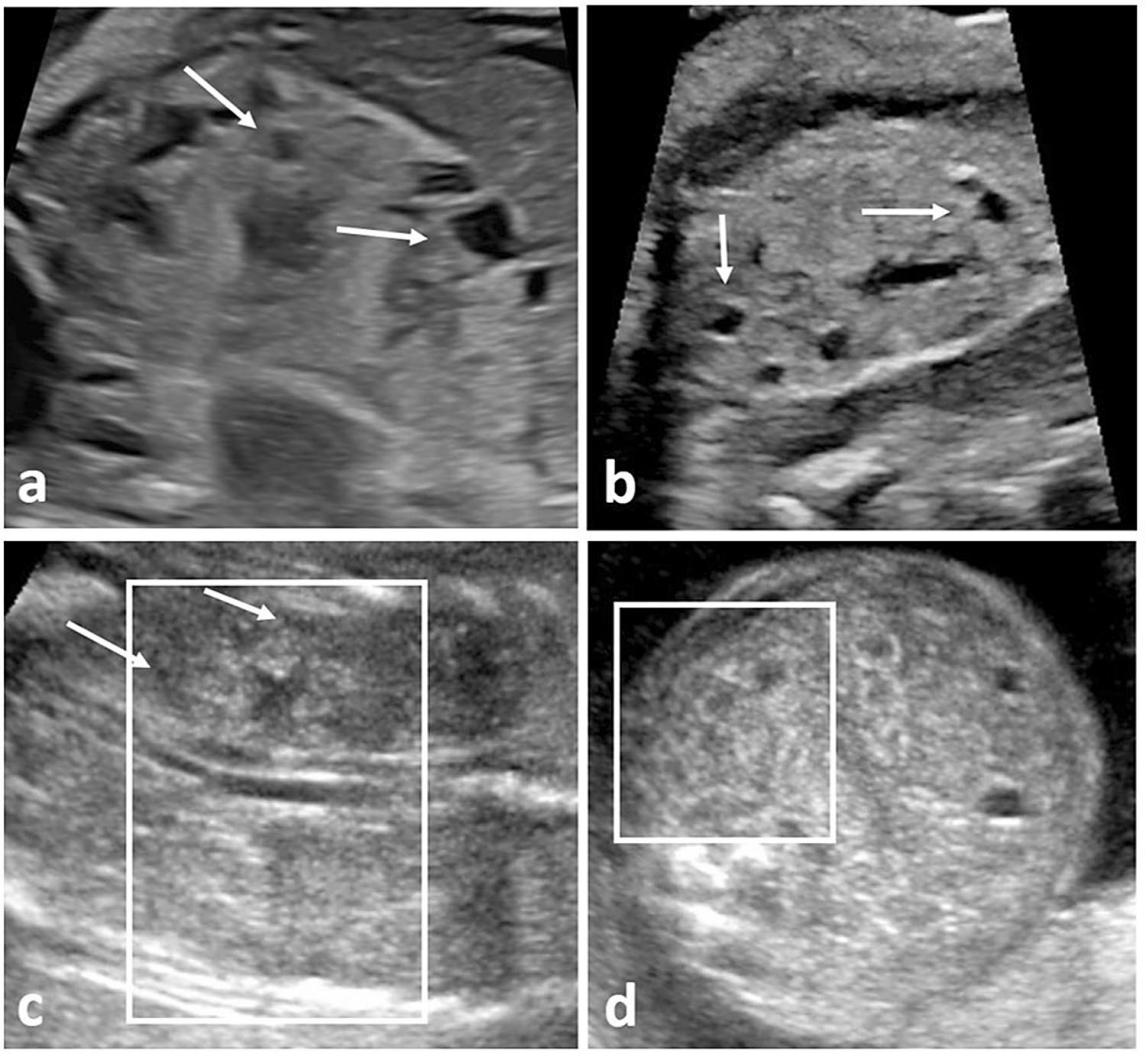
Prenatal ultrasound in fetuses with Mainzer–Saldino syndrome and Beckwith–Wiedemann syndrome (*BWS*). **a**+**b** Show a fetus with Mainzer–Saldino syndrome at 30+3 weeks’ (**a**) and 34+3 weeks’ (**b**): kidneys are enlarged, hyperechogenic, with absent *CMD* and small cortical cysts (white arrows); **c**+**d** show a fetus with BWS at 21+6 weeks’, presenting with absent *CMD* and small cysts (white arrows) located predominantly at the corticomedullary junction (coronary view and axial view – white box)

**Fig. 5:**
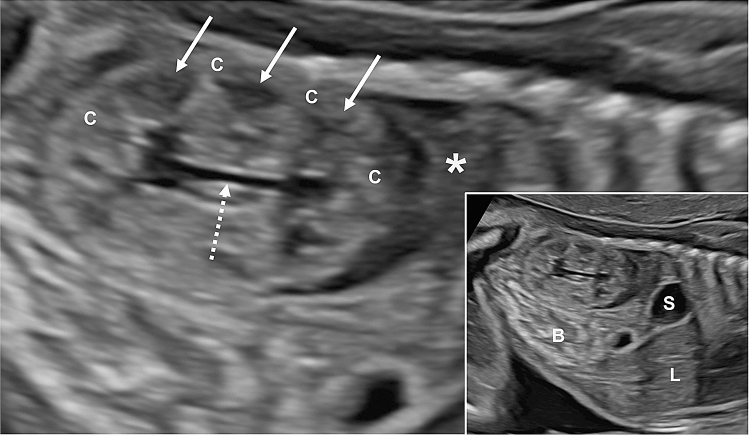
Example of normal midtrimester kidney morphology (longitudinal view) in a fetus at 22 weeks‘. The figure shows a normally sized bean-shaped kidney with  clear demarcation between cortex (C) and medulla: the cortex being less or equally echogenic compared to the liver (L) and the medullary pyramids (white arrows) being less echogenic than the cortex. The renal pelvis (white dotted arrow) is slim. * adrenal gland; *B* bowel; *L* liver; *S* stomach

Table 1 gives information on ultrasound findings with special focus on renal morphology and biometry, and Figs. 1, 2, 3 and 4 illustrate fetal kidney changes in the different diseases. For comparison, Fig. 1 gives an example of normal midtrimester kidney morphology. CMD presented reduced or absent in all fetuses (100%) diagnosed with MKS, BWS, BBS/MKKS, Jeune and Mainzer–Saldino syndrome, in the one fetus with RTD, as well as in most cases of ARPKD (92.3%, Fig. 2a). Reverse CMD (hyperechogenic medulla and hypoechogenic cortex) was only seen in fetuses with ARPKD (7.7%, Fig. 2b), whereas enhanced CMD was a key finding in ADPKD (50%, Fig. 2d) but also in MKS during first and early second trimester (Fig. 3a). Kidneys presented hyperechogenic in all fetuses (100%) suffering from MKS, ADPKD and BBS/MKKS. On ultrasound, multiple kidney cysts were presented in MKS, SGBS, Jeune syndrome and Mainzer–Saldino syndrome (100%, Figs. 3b, c, 4d, 3d, e, 5a, b), BBS/MKKS (66.7%, Fig. 4c) and ARPKD (59.6%, Fig. 2a, b). Scattered or solitary cysts were seen in ADPKD (37.5%, Fig. 2c, e) and in fetuses with Joubert syndrome (40%, Fig. 4a, b). Cysts located at the corticomedullary junction were a unique finding in BWS (Fig. 5c, d). Hydronephrosis was most frequently seen in fetuses with BBS/MKKS (66.7%, Fig. 4c).

**Fig. 6:**
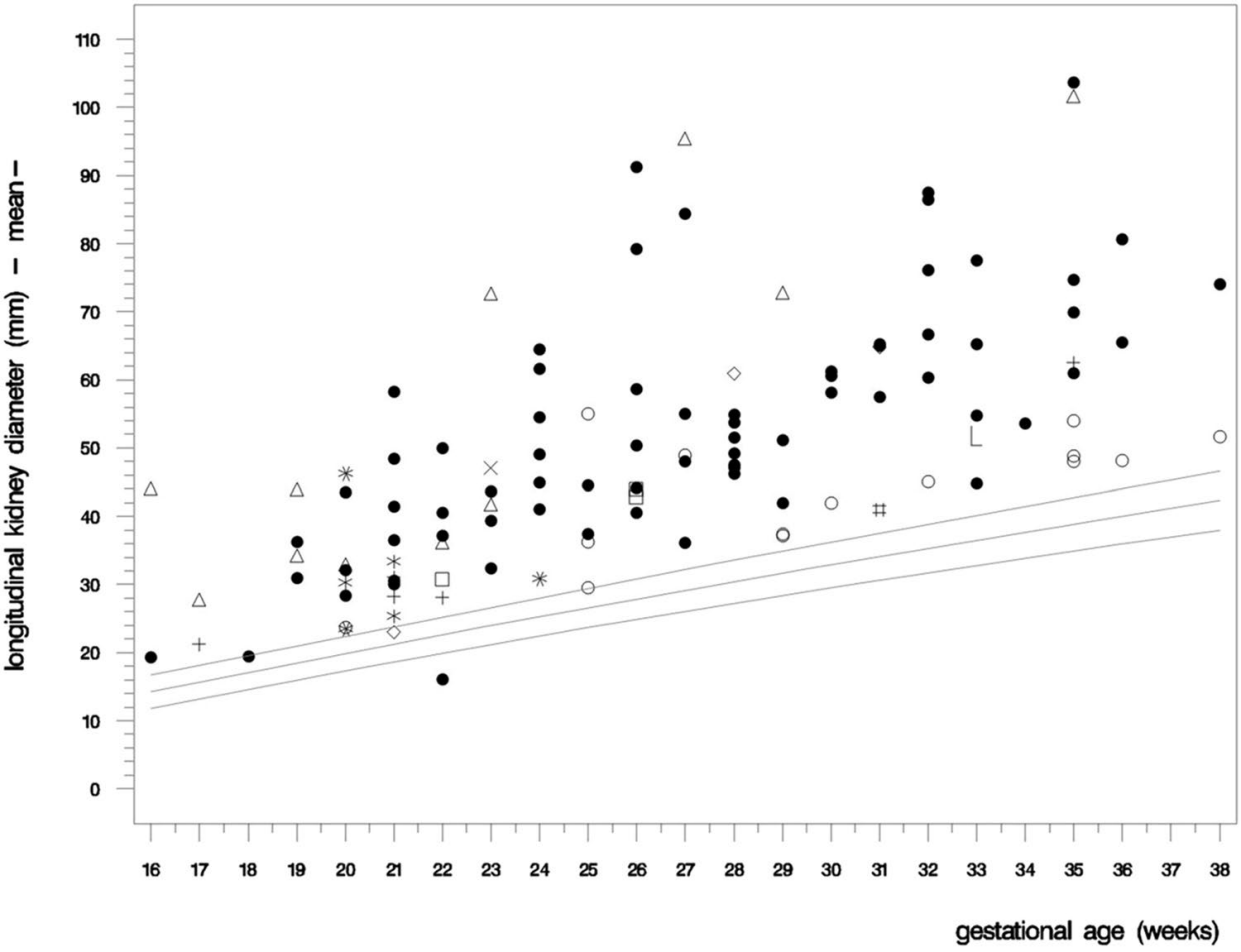
Mean longitudinal renal diameter in fetuses with polycystic kidneys. The longitudinal renal diameter of each case was measured at initial presentation as well as, whenever obtainable, during follow-up scans. Values were collected 150 times in a total of 94 fetuses. For reference values see van [9]; ADPKD (○○○); ARPKD (●●●); BBS/MKKS (□□□); BWS (◊◊◊); Jeune syndrome (×××); Joubert syndrome (∗∗∗); Mainzer-Saldino syndrome (# # #); MKS (△△△); No final diagnosis (***); RTD (∟∟∟); SGBS (+++); *SD* standard deviations; Greatest aberration from expected measurements can be seen in MKS [16.15 ± 9.0 (7.32; 33.32) SD], Jeune syndrome (12.79 SD) and ARPKD [11.64 ± 6.5 (− 3.70; 32.63) SD]; Kidneys of fetuses with *BWS*, unclear diagnosis, *MKKS/BBS* and *RTD* presented enlarged by 9.92 ± 7.74 (1.01; 14.95), 6.77 ± 7.37 (2.09; 15.26), 6.92 ± 1.95 (4.69; 8.32) and 6.66 standard deviations, respectively. Least variation from healthy fetuses were seen in fetuses with *JS* [5.14 ± 2.4 (2.39; 6.98) SD], *SGBS* [5.12 ± 2.7 (3.11; 9.56) SD], *ADPKD* [4.45 ± 4.0 (− 0.24; 15.03) SD] and Mainzer−Saldino syndrome (3.11 SD)

Biometric data of kidneys are shown in Fig. 6. Cases affected by MKS, Jeune syndrome and ARPKD showed the greatest aberration from expected measurements with 16.15 ± 9.0 (7.32, 33.32), 12.79 and 11.64 ± 6.5 (− 3.70, 32.63) standard deviations, respectively, whereas fetuses with JS, SGBS, ADPKD and Mainzer–Saldino syndrome showed the least variation from healthy fetuses with 5.14 ± 2.4 (2.39, 6.98), 5.12 ± 2.7 (3.11, 9.56), 4.45 ± 4.0 (− 0.24, 15.03) and 3.11 standard deviations, respectively [9].

#### Amount of amniotic fluid (AF)

At initial presentation, oligo- or anhydramnios were most frequently seen in fetuses with ARPKD (*n* = 44, 84.6%, mean GA at initial presentation 25 + 2 weeks’, range 16 + 3–38 + 3 weeks’) as well as in the one fetus with RTD (31 + 1 weeks’). Oligo- or anhydramnios was the main reason for referral of patients in all cases of ARPKD diagnosed before 22 + 0 weeks’. We obtained follow-up data on AF in 18 cases (34.6%) of ARPKD: four cases showed initial and persisting anhydramnios; in seven cases, initial oligohydramnios gradually decreased to anhydramnios and in five cases it remained stable in the further course of the pregnancy. In two cases, fetuses initially presented with normal AF, which remained normal in one case and in the other case (initially presenting at 21 weeks’) gradually decreased to anhydramnios by 29 weeks’.

Of the eight fetuses with ADPKD, one initially presented with anhydramnios (at 26 + 2 weeks’), whereas the remaining seven cases (87.5%) presented with normal AF (range 21 + 0–38 + 3 weeks’). Of these seven cases, we obtained follow-up data on AF in four cases: AF remained normal in three cases in the further course of the pregnancy and in one case AF gradually decreased to oligohydramnios.

Of all fetuses with MKS (*n* = 17), 47.1% (*n* = 8) initially presented with either oligo- or anhydramnios (mean GA at initial presentation: 24 + 4 weeks’; range 18 + 5–35 + 3 weeks’). All the remaining cases that presented with normal AF (*n* = 9) were seen before 20 weeks’ gestation (mean GA at initial presentation: 15 + 0 weeks’; range 11 + 1–19 + 4 weeks’), and follow-up on AF is missing in all these nine cases because of early TOP.

Polyhydramnios was a key finding in BWS and SGBS (100 and 66.7%, respectively; Table 1) and persisted in all cases with follow-up data on AF. Fetuses with JS, Jeune syndrome, BBS/MKKS as well as the one fetus with Mainzer–Saldino syndrome all showed normal amounts of AF at initial presentation as well as in follow-up scans, when performed.

#### Other ultrasound findings.

Table 2 gives detailed information on extrarenal ultrasound findings and novel findings have been marked. Skeletal as well as cardiac abnormalities (35.7 and 34.7%, respectively) were the most common findings, followed by abnormalities of the central nervous system (27.6%).

**Table 2:** Other sonographic findings in fetuses with prenatally diagnosed polycystic kidney appearance (*n* = 98)

Diagnosis	Heart	Skeleton	Central nervous system	Urinary bladder	Gastrointestinal system	Other
All (*n* = 98)	All (*n* = 34)	All (*n* = 35)	All (*n *= 26)			
ARPKD (n = 52)	Cardiac hypertrophy (*n* = 12) PE + VSD (*n* = 1) CH + PE (*n* = 4) Pericardial effusion (*n* = 1) ARSA (*n* = 1)	Thoracic hypoplasia (*n *= 19) Micrognathia (*n* = 1) Hexadactyly (*n* = 1)	All (*n *= 6) Ventriculomegaly (*n* = 2) Posterior fossa cyst (*n* = 2) Enlarged posterior fossa (*n* = 1) Megacisterna magna (*n* = 1)	No filling (*n* = 19)	Ascites (*n* = 3)	SUA (*n* = 1) Agenesis of DV (*n* = 1) Nuchal edema (*n* = 1) Hygroma colli and unilateral aplasia of the NB (*n* = 1)
ADPKD (*n* = 8)	Cardiac hypertrophy (*n* = 2)	Thoracic hypoplasia (*n* = 1)	–	–	–	IUGR (*n* = 1) Maternal PKD (*n* = 7)
MKS (*n* = 17)	Cardiac hypertrophy (*n* = 2) Pericardial effusion (*n* = 1) CH + PE (*n* = 1) Unbalanced AV-canal with DORV and hypoplastic aorta (*n* = 1)	Thoracic hypoplasia (n = 4) Hexadactyly (*n* = 8) Rhizomelia (*n* = 2)	Occipital encephalocele (*n* = 13) Anencephaly (*n* = 1) Exencephaly (*n* = 1) Dandy-Walker malformation (*n* = 3)	No filling (*n* = 8)	–	Hydrothorax (*n* = 1)
JS (*n* = 5)	VSD (*n* = 1)	–	Hypoplasia of the entire cerebellum (*n* = 1)^a^ Dandy-Walker malformation (*n* = 4) Occipital encephalocele (*n* = 2) Ventriculomegaly (*n* = 3) pACC (*n* = 2)		–	Hypospadias (*n* = 1)^a^ Exophthalmos (*n* = 1)
MKKS/BBS (*n* = 4)	Pericardial effusion (*n* = 1) AV canal + LPSVC (*n* = 1) Cardiac hypertrophy + LPSVC (*n* = 1)	Hexadactyly (*n* = 3)	–		–	Hydrometrocolpos (*n* = 3)
Jeune syndrome (*n *= 2)	CoA (*n* = 1) VSD (*n* = 1)	Thoracic hypoplasia (*n* = 2) Micromelia (*n* =)	Ventriculomegaly (*n* = 1)		–	Micrognathia (n = 1) Macroglossia (*n* = 1)
Mainzer-Saldino syndrome ( *n *= 1)	Cardiac hypertrophy (*n* = 1)	Micromelia with bent tubular bones (*n* = 1)	Enlarged cisterna magna (*n* = 1)		Liver cysts (*n* = 1)	LGA (*n* = 1) Macrocephaly (*n* = 1)
RTD (*n *= 1)	–	–	–	No filling (*n* = 1)	–	Agenesis of DV (*n* = 1)
SGBS (*n* = 3)	Dextrocardia (*n* = 1)	–	–		–	CDH (*n* = 3)^b^ macRoglossia (*n* = 1) hypospadia (n = 1) Frontal and nuchal edema (*n* = 1)
BWS (*n* = 2)	–	–	–		–	Macroglossia (*n* = 2)
Unknown (*n* = 3)	Dextrocardia (*n* = 1)	Thoracic hypoplasia (*n* = 1) Scoliosis (*n* = 1) Bending of tubular bones (*n *= 1)	Ventriculomegaly (*n* = 1) pACC (*n* = 1) Cerebellar hypoplasia (*n* = 1)	No filling (*n* = 3)	Hyperechogenic bowel (*n* = 1) Gastroschisis (*n* = 1)	–

Abbreviations (in alphabetical order): *ADPKD* autosomal dominant polycystic kidney disease, *ARPKD* autosomal recessive polycystic kidney disease, *ARSA* aberrant right subclavian artery, *AV-canal* atrioventricular canal, *BBS* Bardet-Biedl syndrome, *BWS* Beckwith-Wiedemann syndrome, *CH* cardiac hypertrophy, *CDH* congenital diaphragmatic hernia, *CoA* coarctation of the aorta, *DORV* double outlet right ventricle, DV ductus venosus, *GA* gestational age, *IUGR* intrauterine growth restriction, *JS* Joubert syndrome, *LGA* large for gestational age, *LPSVC* left persistent superior vena cava, *MKKS* McKusick-Kaufman syndrome, *MKS* Meckel-Gruber syndrome (Meckel syndrome), *NB* nasal bone, *pACC* partial agenesis of the corpus callosum, *PE* pericardial effusion, *PKD* polycystic kidney disease, *RTD* renal tubular dysgenesis, *SGBS* Simpson-Golabi-Behmel syndrome, *SUA* single umbilical artery, *VSD* ventricular septum defect

^a^Previously not published in JS

^b^Prevalence of CDH in SGBS has been reported in up to 24% [4]

#### Outcome

Group specific outcomes are given in Tables 1, 3. Of 33 live-born children, 10 suffered neonatal death (NND), which leaves 23 children (69.7%, or 23.5% of the entire cohort) surviving beyond the neonatal period (Table 3). Respiratory insufficiency resulting from pulmonary hypoplasia and pulmonary arterial hypertension was the main cause of neonatal death (*n* = 9, 90%).

**Table 3:** Characteristics of live-born fetuses with prenatally diagnosed polycystic kidney appearance (*n* = 33)

Diagnosis	Amniotic fluid	Sex	GA at delivery	Mode of delivery	Long-term outcome
All (*n* = 33)	Anhydramnios (*n* = 7) Oligohydramnios (*n* = 13) Normal (*n* = 11) Polyhydramnios (*n* = 2)	Male (*n* = 15) Female (*n* = 18)	36 + 0 (24 + 4 – 41–0)	CS (*n* = 20) VB (*n* = 12) Unknown (*n* = 1)	NND (*n* = 10) Survival beyond neonatal age (*n* = 23)
ARPKD (*n* = 22)	Anhydramnios (*n* = 5) Oligohydramnios (*n* = 14) Normal (*n* = 3)	Male (*n* = 10) Female (*n* = 12)	35 + 3 (31 + 2 –38 + 5)	CS (*n* = 12) VB (*n* = 8) Unknown (*n* = 1)	NND (*n* = 9)^c^ Survival beyond neonatal age (*n* = 13)^c^
ADPKD (*n* = 6)	Oligohydramnios (*n* = 1) Normal (*n* = 5)	Male (*n* = 2) Female (*n* = 4)	36 + 5 (24 + 4 – 41 + 0)	CS (*n* = 3) VB (*n* = 3)	Survival beyond neonatal age (*n* = 6)
SGBS (*n* = 1)	Polyhydramnios (*n* = 1)	Male (*n* = 1)	36 + 5	CS (*n* = 1)	NND (*n* = 1)^a^
BBS (*n* = 1)	Normal (*n* = 1)	Female (*n* = 1)	37 + 3	CS (*n* = 1)	Survival beyond neonatal age (*n* = 1)
BWS (*n* = 1)	Polyhydramnios (*n* = 1)	Female (*n* = 1)	35 + 6	CS (*n* = 1)	Survival beyond neonatal age (*n* = 1)
Mainzer–Saldino (*n* = 1)	Normal (*n* = 1)	Male (*n* = 1)	39 + 0	CS (*n* = 1)	Survival beyond neonatal age (*n* = 1)^b^

*ADPKD* autosomal dominant polycystic kidney disease, *AF* amniotic fluid, *ARPKD* autosomal recessive polycystic kidney disease, *BBS* Bardet-Biedl syndrome,* BWS* Beckwith-Wiedemann syndrome, *CDH* congenital diaphragmatic hernia, *CS* cesarean section, *GA* gestational age, *MKKS* McKusick-Kaufman syndrome, *MKS* Meckel-Gruber syndrome (Meckel syndrome), *NND* neonatal death, *SGBS* Simpson-Golabi-Behmel syndrome, *VB* vaginal birth

^a^Death 15 days after birth due to respiratory insufficiency caused by CDH

^b^Death at the age of 5.5 months due to respiratory insufficiency, severe pulmonary hypertension as well as end-stage renal failure

^c^ARPKD: *AF* in 13 children surviving beyond the neonatal period: *n* = 5 with persisting oligohydramnios, *n* = 3 with persisting anhydramnios, *n* = 3 with normal amounts of AF throughout pregnancy, *n* = 2 with oligohydramnios and progressive reduction to anhydramnios; *AF* in nine children that suffered *NND*: *n* = 5 with persisting oligohydramnios, *n* = 3 with persisting anhydramnios, *n* = 1 with normal amounts of AF (death of metabolic derangement 2 days after birth)

## Discussion

**Fig. 7:**
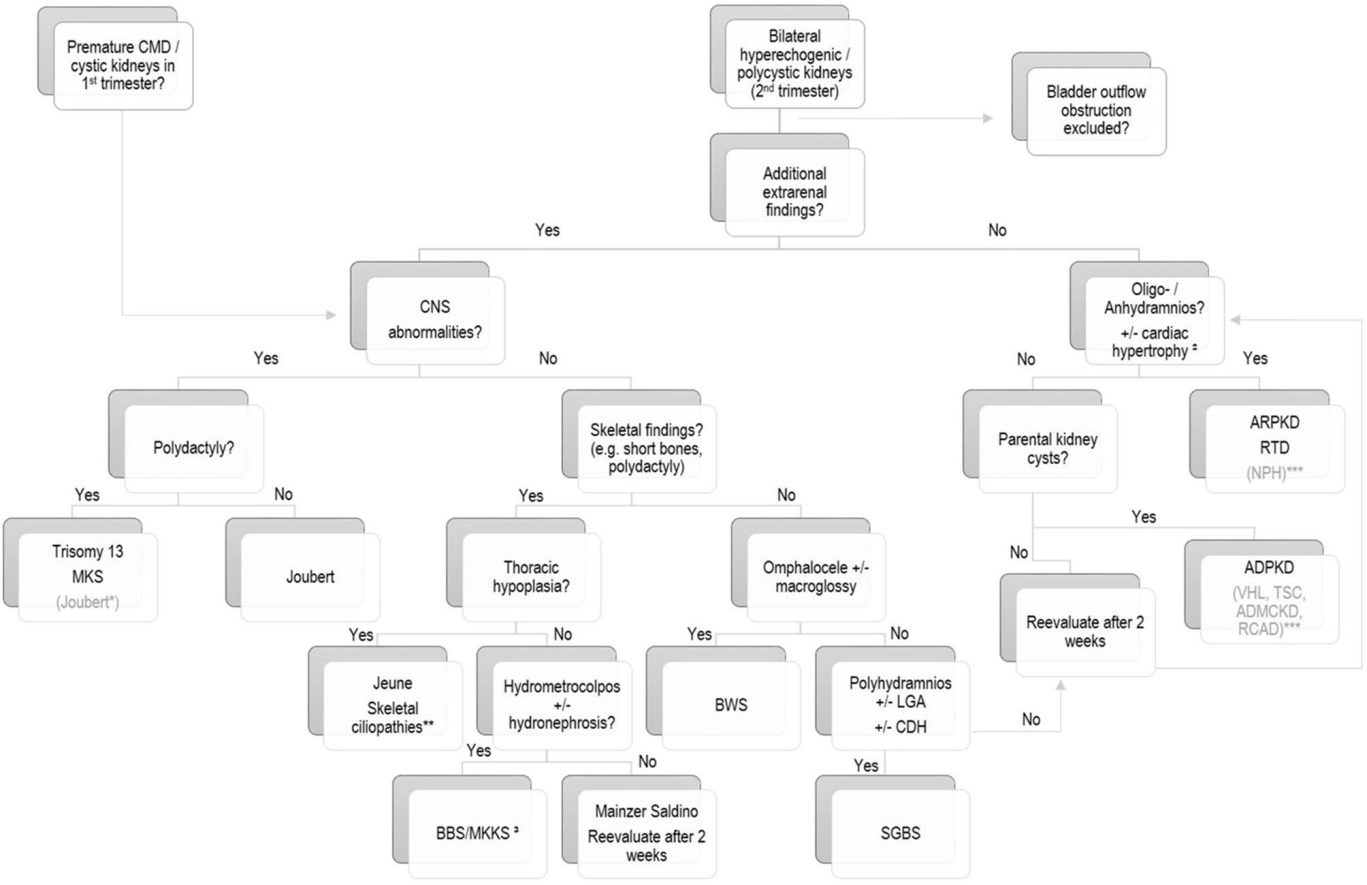
Diagnostic algorithm in the differential diagnosis of bilateral polycystic and/or hyperechogenic kidneys on prenatal ultrasound, modified from *Erger et al.* [1] and *Ortiz-Brüchle et al. *[27]. *ADMCKD* autosomal dominant medullary cystic kidney disease, *ADPKD* autosomal dominant polycystic kidney disease, *ARPKD* autosomal recessive polycystic kidney disease, *BBS* Bardet–Biedl syndrome, *BWS* Beckwith–Wiedemann syndrome, *CMD* corticomedullary differentiation, *CNS* central nervous system, *CDH* congenital diaphragmatic hernia, *LGA* large for gestational age, *MKKS* McKusick–Kaufman syndrome, *MKS* Meckel–Gruber syndrome, *NPH* nephronophthisis, *RCAD* “renal cysts and diabetes” syndrome, *RTD* renal tubular dysgenesis, *SGBS* Simpson–Golabi–Behmel syndrome, *SRP* short rib polydactyly syndromes, *TSC* tuberous sclerosis, *VHL* von Hippel–Lindau syndrome, ²characteristic features of the “fetal cardiorenal syndrome” (*Merz WM et al*. [10]), ³ also check for structural heart defects!, *Polydactyly, although rarely, has been reported in Joubert syndrome, ** including Ellis–van Creveld syndrome (*EVC*, chondroectodermal dysplasia), Sensenbrenner syndrome (*SS*, cranioectodermal dysplasia), and short-rib polydactyly syndromes (SRPSs) types I–IV, ***prenatal manifestation is very rarely reported

The most indicative diagnostic signs in fetuses with polycystic kidney appearance include bilateral kidney enlargement and hyperechogenicity of the renal parenchyma, the presence and location of cysts and changes of CMD, together with the amount of amniotic fluid and presence of extrarenal ultrasound findings. Based on studies of *Ortiz-Brüchle et al.* [27] as well as *Erger et al.* [1] and our new findings, we aimed to modify and, in part, expand the diagnostic algorithm (Fig. 7) to help differentiate between the most frequent underlying diseases by prenatal ultrasound.

### Renal changes in different diseases

In ARPKD, kidneys of fetuses usually show hyperechogenic parenchyma, in the vast majority with absent or, less frequently, reversed CMD [1, 11]. Cysts are usually a few millimeters in size and only partially visible on ultrasound [12, 13]. Interestingly, 60.6% of affected fetuses in our study showed cysts on prenatal ultrasound, which is a significantly higher proportion than reported before [1, 11]. Progression in size and more successful detection of cysts in higher gestational ages and with the use of high-resolution ultrasound transducers could be observed in our cohort and has been reported before [11, 14]. In ADPKD, kidneys may present enlarged and sometimes with rather increased CMD. Cysts are less frequently observed prenatally in ADPKD (37.5% in our study) and usually reach visible size on ultrasound only postnatally [2]. Cysts in ADPKD are true closed cysts that continue to increase in number and in size throughout life. Progression in kidney volume in patients with ADPKD correlates with decline in kidney function [15].

Assessment of CMD on prenatal ultrasound is usually not possible before 19 weeks’. However, in fetuses with MKS, examinations during 1st and early 2nd trimester revealed a CMD that appeared premature or “too advanced” for the gestational age, which might be attributed to cystic remodeling, especially of the renal medulla. Eventually, this leads to distinctly enlarged and hyperechogenic kidneys and, eventually, absent CMD in the further course of the pregnancy. Cysts are usually of small diameter (but mixed types are possible), leading to a “mottled” appearance of the renal parenchyma [2, 6, 15].

Renal changes in fetuses with Joubert syndrome (enlarged, hyperechogenic, cystic kidneys with reduced or absent CMD) are found in approximately 25–30% [2, 8, 15] and dependent on the underlying mutations [16]. In Jeune syndrome, renal abnormalities were present in approximately 1/3 of cases and mainly include cysts or hydronephrosis [8, 17].

In BWS, renal changes (kidney enlargement, abnormalities of the collecting system, cysts, nephrocalcinosis and/or nephrolithiasis) are seen in up to 59% of patients and are frequently accompanied by polyhydramnios and fetal macrosomia. Patients with UPD (chromosome 11p15 paternal uniparental disomy) and patients with IC1 gain of methylation are more prone to renal changes (26.4 and 32.5%, respectively, confirming our findings) than patients with IC2 hypomethylation (8.9%) [4, 18].

Differential diagnosis between BBS and MKKS remains difficult before the age of 5 years. Renal changes (hyperechogenicity, absent CMD, cystic changes, developmental abnormalities) are one of the main criteria of BBS and affect 53–82% of patients [6, 19, 20]. In MKKS, renal disease occurs less frequently and is often secondary to genital malformations. Common renal changes include cyst formation, absent CMD as well as hydronephrosis [8, 19]. SGBS is characterized by visceral enlargement, often also involving the kidneys, and polyhydramnios is seen frequently. Somewhat less frequently, kidneys show morphological changes, such as hyperechogenicity of the parenchyma or cysts. Although said to often appear only postnatally, all three fetuses in this study already showed corticomedullary cysts and absent CMD on prenatal ultrasound [4, 21]. Kidney changes in fetuses with RTD are subtle: kidneys can present mildly enlarged, hyperechogenic with reduced or absent CMD and oligohydramnios usually precedes these changes by weeks (onset usually around 20–22 weeks’) [5].

### Time of diagnosis

Consistent with previous findings, kidney abnormalities in our cohort usually did not become apparent until the 2nd trimester [1, 2, 4, 8, 22]. In BBS/MKKS, Mainzer–Saldino syndrome and RTD, abnormalities presented even later in pregnancy. First trimester diagnosis can be achieved in MKS, especially when there’s a history of a previously affected pregnancy [23]. Maternal medical history of ADPKD may lead to earlier diagnosis of ADPKD in the fetus, however, possibility of de-novo mutation as well as unknown maternal ADPKD (as shown in one of our cases, each) should be kept in mind and highlights the importance of detailed anamnesis.

In ARPKD oligo- or anhydramnios is still the main reason for referral, resulting in a diagnosis rate of only 32.7% of all ARPKD cases in our cohort before 22 weeks’ gestation. Poor diagnosis rate might in part be explained by the fact that ultrasound assessment of amniotic fluid volume is an obligatory part of the regular check-ups at the patients’ gynecologists, whereas assessment of the fetal kidneys is not [24].

### Renal biometry

Fetuses with MKS and ARPKD in late second and early third trimester showed the highest degree of kidney enlargement, which is consistent with previous studies. In MKS, kidneys appeared enlarged already during 1st and early 2nd trimester, however, there is a lack of comparative values before 15 weeks’ of gestation [1, 2, 9]. Especially the longitudinal renal diameter has been found particularly suitable in the assessment of fetal kidney enlargement [7]. Interestingly, in our cohort both two fetuses with Jeune syndrome showed significantly enlarged kidneys, which has not been reported before.

### Amniotic fluid (AF) volume

Reduced AF represents a frequent but not obligatory diagnostic criterion of ARPKD [11]. At initial presentation, 84.6% of fetuses with ARPKD showed oligo- or anhydramnios in our study, which is slightly higher than reported before (69–82%) [1]. In ARPKD, AF can further decrease during pregnancy or remain stable [25]. In contrast, fetuses with prenatal renal manifestations of ADPKD usually show normal amounts of AF [1, 2] without tendency of progressive AF reduction [25]. Similar to ARPKD, oligo- and anhydramnios from second trimester onwards are typical for RTD [5] and MKS [1, 23]. Fetuses with JS, Jeune syndrome and MKKS/BBS usually show normal AF [2, 8], whereas fetuses with overgrowth syndromes tend to show higher occurrence of polyhydramnios [2, 4].

In our cohort of live-born children with ARPKD, a comparable proportion of both infants surviving (84.6%) or not surviving beyond neonatal age (88.9%) had decreased AF volume in utero (Table 3). Thus, the presence of oligo- or anhydramnios during pregnancy did not necessarily predict a lethal course. Our study findings also showed that normal amounts of AF did not always guarantee survival, as one case of ARPKD and normal amounts of AF throughout pregnancy died of metabolic derangement 2 days after birth. Serial amnioinfusion for cases of anhydramnios due to congenital nephropathy is still viewed controversial and studies investigating a potential benefit still in process [26].

### Other sonographic findings

The presence of, in part, typical extrarenal abnormalities in fetuses with hyperechogenic and polycystic kidneys has been shown in the past to help differentiate between different underlying diseases. Figure 7 illustrates a previously described diagnostic algorithm [1, 27] which we aimed to expand by our ultrasound and clinical findings.

### Strengths and limitations

Strengths of our study are the high number of cases as well as a great variety of diseases showing the wide span of differential diagnoses of fetal polycystic kidneys. Aside from general limitations of retrospective studies, the small numbers of cases in certain disease groups as well as the restrictions associated with tertiary referral centers, which tend to see more severe cases and may therefore have higher rates of TOP as well as lower overall survival, are limitations of this study. Especially in ARPKD, prenatal diagnosis has been described to be associated with significantly increased morbidity and mortality [1, 11]. Due to the time frame of the study, genetic testing was not always performed according to current standards.

In this study, we present one of the largest series of prenatally diagnosed polycystic appearing kidneys. Our workup of the specific morphology of kidney changes as well as extrarenal findings on prenatal ultrasound adds valuable data to preexisting studies on genotype–phenotype correlations in different diseases. However, the sonographic overlap remains challenging. We conclude that gestational age at manifestation, kidney size, visibility of cysts, echogenicity, amniotic fluid volume and the presence of associated extrarenal malformations allow to differentiate between the most frequent underlying diseases by following a diagnostic algorithm. In doing so, more targeted genetic testing and multidisciplinary counseling of parents can be achieved.
